# Cementless bipolar hemiarthroplasty in femoral neck fractures in elderly

**DOI:** 10.4103/0019-5413.80042

**Published:** 2011

**Authors:** SKS Marya, R Thukral, R Hasan, M Tripathi

**Affiliations:** Max Institute of Orthopedics and Joint Replacement, Max Super Specialty Hospitals, Saket, New Delhi, India

**Keywords:** Cementless bipolar, femoral neck fractures, comorbidities, elderly

## Abstract

**Background:**

Cemented hip arthroplasty is an established treatment for femoral neck fracture in the mobile elderly. Cement pressurization raises intramedullary pressure and may lead to fat embolization, resulting in fatal bone cement implantation syndrome, particularly in patients with multiple comorbidities. The cementless stem technique may reduce this mortality risk but it is technically demanding and needs precise planning and execution. We report the perioperative mortality and morbidity of cementless bipolar hemiarthroplasty in a series of mobile elderly patients (age >70 years) with femoral neck fractures.

**Materials and Methods:**

Twenty-nine elderly patients with mean age of 83 years (range:71-102 years) with femoral neck fractures (23 neck of femur and 6 intertrochanteric) were operated over a 2-year period (Nov 2005–Oct 2007). All were treated with cementless bipolar hemiarthroplasty. Clinical and radiological follow-up was done at 3 months, 6 months, 12 months, and then yearly.

**Results:**

The average follow-up was 36 months (range 26-49 months). The average duration of surgery and blood loss was 28 min from skin to skin (range, 20–50 min) and 260 ml (range, 95–535 ml), respectively. Average blood transfusion was 1.4 units (range, 0 to 4 units) Mean duration of hospital stay was 11.9 days (7–26 days). We had no perioperative mortality or serious morbidity.

We lost two patients to follow-up after 12 months, while three others died due to medical conditions (10–16 months post surgery). Twenty-four patients were followed to final follow-up (average 36 months; range: 26–49 months). All were ambulatory and had painless hips; the mean Harris hip score was 85 (range: 69–96).

**Conclusion:**

Cementless bipolar hemiarthroplasty for femoral neck fractures in the very elderly permits early return to premorbid life and is not associated with any untoward cardiac event in the perioperative period. It can be considered a treatment option in this select group.

## INTRODUCTION

The objective of treatment of femoral neck fractures in the mobile elderly population is early restoration of premorbid walking ability and quality of life.^1^–^3^ Internal fixation by dynamic hip screw or proximal femoral nail are often unsuccessful as unacceptably high rates of failure (avascular necrosis, nonunion, and repeat surgical procedures) are known to occur.^3^–^4^ In the young the emphasis is on bone stock preservation, but in the elderly return to premorbid status with early mobilization is paramount.^3^

Hemi- or total hip arthroplasty is an accepted treatment of fracture neck of femur in the elderly.^1^–^2^ Cemented prostheses have been used with high success rates^1^,^5^,^6^ but are associated with high perioperative morbidity (hemodynamic instability, cardiopulmonary complications, etc.) and mortality.^4^,^7^ Cementless stems avert this so-called ‘cement reaction’ or bone cement implantation syndrome (BCIS),^2^,^7^ though there have been many complications noted with this technique (for example, intraoperative and immediate postoperative fractures, loosening and subsidence, with thigh pain etc).^2^,^5^ The advantages of the cemented technique seem to be offset by its mortality risk and the advantages of the cementless option by its increased morbidity. Thus, in some of these very elderly morbid patients the orthopedic surgeon is faced with a dilemma regarding the correct surgical choice, which should be one that can promise pain relief and rapid resumption of function and, at the same time, prevent mortality and reduce morbidity.

We report a retrospective analysis of a series of 29 consecutive cases of femoral neck fractures in the very elderly population (age >70 years) with associated multiple comorbidities treated by cementless hemiarthroplasty by a single surgeon over a 2-year period and present their perioperative morbidity and mortality and early postoperative results.

## MATERIALS AND METHODS

**Figure 1:**
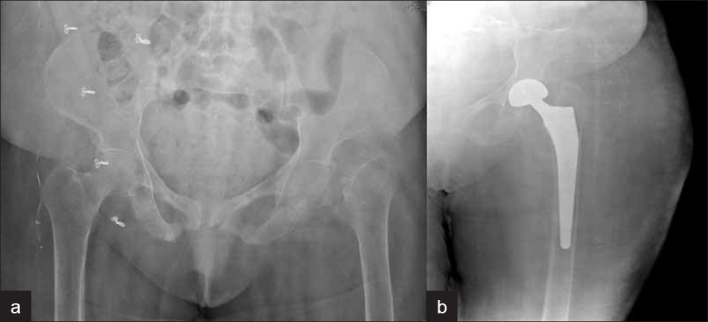
Radiographs (anteroposterior view) of 83-year-old patient with fracture neck of femur on left side. (a) Preoperative; (b) 24 months after cementless bipolar hemi-replacement

**Figure 2:**
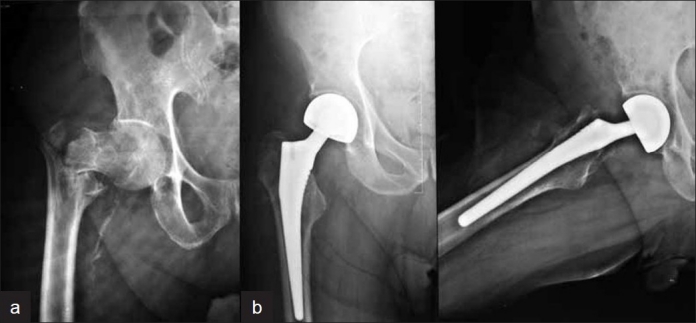
Preoperative radiographs of right hip (anteroposterior view) of 73-year-old patient shows (a) unstable intertrochanteric fracture of the femur; (b) radiograph (anteroposterior and frog leg view) 24 months after cementless bipolar hemi-replacement

Between November 2005 and October 2007, a single operating surgeon performed 29 consecutive cementless hemiarthroplasties for cervico-trochanteric femoral fractures in 29 patients, 18 males and 11 females. These patients, with a mean age of 83.8 years (range: 71-102 years), presented with displaced intracapsular fracture neck of femur, Garden type III/IV (*n*=23) [Figure 1a], or comminuted intertrochanteric fracture (*n*=5) [Figure 2a]; one patient presented with a 7-month-old intertrochanteric fracture and failed fixation with a dynamic hip screw plate. All but one of the patients had been ambulatory, either without support (*n*=19) or with support (cane or walker) (*n*=9), before the injury. We did not conduct any bone mineral density studies in these patients as all were above the age of 70 years and on prolonged medication for chronic medical conditions; also, all but one (failed intertrochanteric fixation) presented to us with fractures following trivial falls at home.

All patients had multiple comorbidities. Twenty-three patients were hypertensive (on irregular treatment), twelve had coexistent diabetes mellitus, and fifteen had coronary artery disease. Twenty-three patients had one or more of the following: benign prostatic hypertrophy (BPH), carcinoma prostate, carcinoma colon, carcinoma intestine, intestinal obstruction, urinary tract infection (UTI), chronic renal failure (CRF), Alzheimer disease, transient ischemic attack (TIA), stroke, dementia, glaucoma, impaired vision, anemia, hypothyroidism, depression, psychosis, chronic bronchitis, asthma, recurrent pleural effusion, recent septicemia, or an associated extremity fracture [Table 1]. As such, all patients fell into ASA grades III (10 patients) and IV (19 patients) (associated with moderate to severe risk, functional limitations, and threat to life) risk for surgery.

**Table 1:** Clinical details of patients

ID no.	Age/ Sex	Primary diagnosis	Premorbid ambulatory status	Comorbidity				Op time (min)	Blood loss (ml)	Hospital stay (days)	Periop morbidity details	Full weightbearing (weeks)
				DM	HTN	CAD	Other medical conditons					
B01	88 M	NOF	No support	Y	Y	Y	-	24	340	12	Lengthening (1.5 cm)	2 (disch)
B02	77 M	NOF	No support	Y	N	Y	Ca prostrate	25	360	8	Uneventful	6
B03	102 M	NOF	No support	N	N	N	BPH, anemia, bronchitis	27	390	17	Uneventful	6
B04	86 F	NOF	Walker	N	Y	N	Hypothyroid, Alzheimer	28	150	9	Uneventful	6
B05	83 F	IT	No support	N	Y	Y	UTI, anemia	26	520	9	Uneventful	6
B06	84 F	NOF	No support	Y	Y	Y	-	25	200	11	Uneventful	6
B07	83 M	NOF	Walker	Y	Y	Y	UTI, CRF, BPH, recent septicemia, distal humerus fracture	24	185	18	Uneventful	12
B08	82 M	NOF	No support	Y	Y	Y	-	23	115	14	Uneventful	6
B09	85 M	NOF	No support	Y	Y	N	BPH	28	270	13	Uneventful	6
B10	80 M	NOF	Cane	Y	Y	Y	TIA	26	320	11	Uneventful	6
B11	85 M	NOF	No support	Y	Y	Y	Stroke	24	170	13	Uneventful	6
B12	97 M	NOF	No support	N	Y	N	Bronchitis, anemia	27	235	7	Uneventful	6
B13	74 M	NOF	No support	Y	Y	N	Hypothyroid	20	185	18	Uneventful	6
B14	88 F	NOF	No support	N	Y	N	Hypothyroid	25	220	7	Uneventful	6
B15	84 M	NOF	Cane	N	N	Y	Depression	24	115	12	Lengthening (1.5 cm)	2 (disch)
B16	87M	IT	Walker	N	N	Y	BPH, glaucoma, right eye blindness	23	535	11	Uneventful	6
B17	76F	NOF	No support	N	N	N	UTI, anemia, psychosis	26	165	7	Uneventful	6
B18	87F	NOF	Walker	N	N	N	Dementia, asthma, infected sacral bedsore	28	245	19	Uneventful	6
B19	82F	NOF	No support	N	Y	Y	Ca colon	30	490	8	Uneventful	6
B20	78M	NOF	No support	N	Y	Y	-	26	290	12	Uneventful	6
B21	92M	IT	Walker	Y	Y	Y	UTI, recurrent pleural effusion, Ca prostrate with brain secondaries, anemia	31	245	12	Post-op ICU (dyselectrolytemia)	6
B22	80M	NOF	No support	Y	Y	Y	-	42	165	10	Uneventful	6
B23	71F	7-monthold failed IT	Bedridden	Y	Y	N	Ca. small intestine – operated	50	225	8	Uneventful	6
B24	84M	NOF	No support	N	Y	N	BPH, TIA	39	280	8	Uneventful	6
B25	90M	IT	No support	N	Y	N	BPH, subacute intestinal obstruction	35	160	26	Postop ICU (dyselectrolytemia)	6
B26	84M	NOF	No support	N	Y	N	Chronic bronchitis, BPH, depression	27	95	11	Uneventful	6
B27	81F	NOF	Cane	N	Y	N	Dementia, operated bilateral TKA	20	200	8	Uneventful	6
B28	73F	IT	No support	N	Y	N	Asthma, hypothyroid	35	425	8	Uneventful	6
B29	88F	NOF	Walker	N	Y	Y	-	29	245	19	Postop ICU (pulmonary edema)	6

NOF: Fracture neck of femur, IT: Fracture intertrochanteric femur, disch: Discharge, BPH: Benign prostatic hypertrophy, UTI: Urinary tract infection, TIA: Transient ischemic attack, TKA: Total knee arthroplasty, CRF: Chronic renal failure, Ca:Carcinoma, ICU: Intensive care unit

Combined spinal-epidural anesthesia was given to all patients. We used the anterolateral approach, with anterior hip dislocation. Bipolar hemi-replacement was performed with the cementless extensively hydroxyapatite-coated Corail^™^ stem (DePuy, USA) (size 9-14) and Hastings^™^ modular bipolar cup (DePuy, USA) (size 39-53). The stability in the axial and rotational plane was assessed before definite insertion of the femoral stem.

Prophylactic intravenous antibiotics were used for 2 days in all patients, followed by oral antibiotics for further 5 days. All patients were kept on physical (ankle pumps) and chemical prophylaxis for DVT (deep vein thrombosis) during their hospital stay. The epidural catheter was removed on 3^rd^ post-operative day (POD). Side turning in bed was allowed immediately. Bedside sitting was started on the 1^st^ postoperative day (POD) and patients were made to stand on the 2^nd^ POD, toe touch with walker was started on 3^rd^ POD and walking upto toilet was allowed on 5^th^ POD (at time of discharge), with progression to full weight bearing at 6 weeks in most of the patients.

Patients were reviewed postoperatively at 2 weeks (for removal of staples), 6 weeks, 3 months, 6 months, 12 months, and then yearly. Patients were clinically and radiographically evaluated at each follow-up. Harris hip scores and pain scoring (using the visual analog scale) were used as clinical outcome measures. Radiological evaluation included standard anteroposterior and lateral radiographs at 3 months, 6 months, 12 months [Figures 1b and 2b], and then yearly for evidence of stem subsidence, lysis, or loosening, as well as to look for acetabular erosion or protrusion or heterotopic ossification.

## RESULTS

The average duration of surgery and blood loss was 28 min from skin to skin (range, 20–50 min) and 260 ml (range, 95–535 ml), respectively. Average blood transfusion was 1.4 units (range, 0 to 4 units) Mean duration of hospital stay was 11.9 days (7–26 days). We had no perioperative mortality or serious morbidity. Two patients suffered with subacute intestinal obstruction leading to dyselectrolytemia; both had to be nursed in (ICU) intensive care unit for 5 days before discharge. One patient developed fluid overload (with basal pulmonary edema) and was also kept in ICU postoperatively.

Two patients had lengthening (~1.5 cm). Ambulation was delayed in two patients, one due to associated elbow fracture and the other due to generalized weakness. The remaining 25 patients were discharged on toe-touch-weight-bearing walking with walker support. Partial to full weight-bearing was permitted only at 6 weeks. All of these patients achieved preinjury ambulatory status at 3 months. We had no instances of infection or dislocation.

We lost two patients to follow-up after 12 months, while three others died due to medical conditions (10–16 months post surgery). Twenty-four patients were followed to final follow-up (average 36 months; range: 26–49 months). All were ambulatory and had painless hips; the mean Harris hip score was 85 (range: 69–96) [Table 2].

**Table 2:** Results at 3 months, 12 months, and final follow-up (average 36 months)

ID no.	Total follow-up (in months)	Pain (VAS) (3 months)	Harris hip score (3 months)	Harris hip score (12 months)	Harris hip score (final follow-up/at 36 months)
B01	49	2	69	89	89
B02	48	2	62	75	72
B03	Died (at 16 months)	1	71	89	-
B04	46	1	65	75	71
B05	44	3	72	91	89
B06	44	1	75	91	89
B07	Lost to follow-up (>12 months)	0	61	75	-
B08	42	0	71	89	89
B09	42	1	89	94	91
B10	41	2	71	86	86
B11	40	2	75	89	89
B12	39	0	89	84	84
B13	38	1	91	94	94
B14	37	1	89	93	93
B15	36	1	75	90	90
B16	35	0	57	75	75
B17	35	0	94	96	96
B18	34	0	51	69	69
B19	Died (at 10 months)	1	72	-	-
B20	31	0	91	93	93
B21	Died (at 10 months)	2	57	-	-
B22	30	0	75	86	86
B23	30	0	69	75	71
B24	29	0	71	93	93
B25	28	1	57	86	86
B26	Lost to follow-up (>12 months)	2	89	91	-
B27	27	1	58	69	69
B28	27	2	89	94	94
B29	26	2	48	82	82

VAS: Visual analogue score

There was no incidence of stem subsidence, acetabular erosion, acetabular protrusion, or heterotopic ossification in any of the patients during the study period. None of the patients have needed revision or conversion to total hip replacement to date. At last follow-up, patients with intra- and extracapsular fractures had similar clinical results though, radiologically, patients with trochanteric fractures continued to demonstrate displaced trochanteric fragments (as on the immediate postoperative films).

## DISCUSSION

The treatment of femoral neck fractures in the mobile elderly is directed at rapid restoration of preinjury functional and ambulatory status.^1^–^3^ As intra- and extracapsular fractures are separate entities with different prognoses, we discuss them one after the other.

Algorithms for femoral neck fracture treatment have been defined. Leighton *et al*.^3^ recommend prosthetic replacement for patients more than 60 years old and having a displaced femoral neck fracture.^3^ Unipolar or bipolar (cemented) hemiarthroplasty has shown the most reliable and predictable outcomes. Uncemented stems are to be considered in patients with significant cardiovascular risk factors and total hip arthroplasty in the ‘active elderly patient,’ while unipolar prostheses (Moore or Thompson) are used only in medically infirm, minimally ambulatory patients.^3^

Literature abounds with success stories with the use of cemented bipolar hip replacements in the early stage; it is reported to be associated with relatively few complications and low mortality rates.^5^ Periprosthetic femoral fractures have been reported with uncemented hemiarthroplasty.^1^,^5^ Elderly frail patients tolerate bone cement as it reinforces osteoporotic proximal femurs.^1^–^3^,^5^

Postoperative mortality following hip replacements is usually due to cardiopulmonary causes (myocardial infarction or pulmonary emboli).^1^ Intraoperative deaths (cardiac arrest) during hip arthroplasty occur infrequently and have been associated with bone cement (BCIS).^7^,^8^ Patients with severe underlying cardiovascular disease are more prone to this problem.^7^ The hemodynamic effects of medullary fat embolism during the process of cement pressurization — rather than the toxic effects of the cement itself — cause BCIS.^8^–^9^ The syndrome manifests with acute pulmonary hypertension, right ventricular dysfunction, myocardial ischemia, hypotension, and even sudden death.^7^,^9^–^10^ Severity does not correlate with the amount of cement used. Rarely, this syndrome may occur in the absence of methyl methacrylate use.^2^,^10^ As reported by Lo and Chen, cemented replacements require relatively more time and have more blood loss as compared to cementless replacements.^5^ In our series, the average operative time was 28 min (range: 20–50 min) and average blood loss 260 ml (range: 95–535 ml). There was no intraoperative or immediate postoperative mortality. This may be due in part to the excellent postoperative care and intensive care setup we had available. However, we have assessed our results without standardizing the different variables responsible (viz. age, gender, fracture classification, osteoporosis, etc.), and this may be one of the drawbacks of our study.

A Cochrane database survey,^1^ with 17 trials involving 1920 patients, confirmed that with cemented prostheses patients had less pain, better mobility, and no significant difference in complications compared with cementless prostheses patients, at a mean follow-up of 1 year. Similarly, no significant differences were found between unipolar and bipolar hemiarthroplasty (seven trials, 857 participants, 863 fractures).^1^ Dorr *et al*.^2^ also, in his prospective study of treatment of displaced femoral neck fractures, found no differences in pain, ambulation, or aids required between any of the femoral stem fixation methods. They advised against the use of cementless femoral stems in wide canals (Dorr type C and D femurs) due to higher instances of subsidence and loosening as a result of inadequate press-fit.^2^

Non-cemented arthroplasty produces lower intramedullary pressures, fewer emboli, and much less hemodynamic disturbance.^2^ Transesophageal echocardiography studies show that cemented stems produce greater and more prolonged embolic cascades,^11^ and that the emboli are of greater number and size and duration of the cascades is longer.^7^ One option to reduce these cardiopulmonary complications (including BCIS) is the use of modern cementing techniques, appropriate anesthesia interventions, and adequate patient preparation. Another option is use of the cementless technique.^1^,^7^,^12^ As demonstrated in our study, a technically correct cementless femoral stem technique can virtually eliminate the mortality, and cause significantly less complications (as with cemented arthroplasty).

On the other hand, the ideal treatment for intertrochanteric fracture in the very elderly is debatable. Unstable intertrochanteric fractures are historically associated with a high rate of complications.^4^ Immediate partial or full weight-bearing in this patient group is crucial – though not always possible – after internal fixation with dynamic hip screws or proximal femoral nails. Cutting-out of these hip screws has been reported in 4%-20% of cases.^4^ Primary total hip replacement has been considered a viable option in a select group of previously independent mobile patients and is reported to be associated with significantly lower complication rates.^4^

Another factor contributing to this paradigm shift in treatment is the presence of multiple comorbidities. The presence of four or more comorbidities has been shown to increase the risk of death by approximately 78%.^13^–^15^ Rodop *et al*.^14^ published a study on standard hemiarthroplasty for the treatment of displaced intertrochanteric fractures in a small group of 54 elderly patients; they reported good functional results in terms of walking ability of their patients. Similarly, Haentjens *et al*.^16^ found better functional outcome and reduced morbidity (pressure sores, pulmonary infection, and atelectasis) with arthroplasty, but mortality rates were not reduced.^16^ Cemented prostheses have been used routinely, and these usually provide immediate stability and permit full weight-bearing.^7^,^9^

Kim *et al*.^17^ compared the 2-year results of long-stem cementless calcar-replacement hemiarthroplasty with the results after proximal femoral nail for unstable intertrochanteric fractures in 58 elderly patients. A superior clinical outcome (with regard to hospital stay, time to weight-bearing, or general complications) was seen with proximal femoral nails. However, there was no advantage in functional outcome compared to the arthroplasty group.^17^ Dislocation has been a major complication with total hip replacement after comminuted intertrochanteric fractures,^16^ and bipolar arthroplasty has been shown to reduce this risk.^6^,^13^,^16^

Primary cemented arthroplasty for intertrochanteric fractures is technically challenging. In severely comminuted fractures, restoration of limb rotation and length can be demanding.^4^ Moreover, dislocation is a very real possibility and can seriously compromise results. To prevent extrusion, all loose fragments, including the greater and the lesser trochanters, need to be attached with cerclage wires or strong non-absorbable sutures before cementing the stem.

The debate then shifts to whether cementless hip arthroplasty in the very elderly is successful or not, given the possible complications noted with improper technique (e.g., fractures, loosening, stress shielding, thigh pain, subsidence, etc.). Uniformly good results have been reported with cementless total hip arthroplasty in the very elderly population.^6^,^18^–^20^ Some studies have even shown less operating time and blood loss for the uncemented cohorts,^20^ with no worse postoperative mortality or complication rates.^20^ Cementless implantation has been criticized for possible component instability and inadequate osteointegration due to the poor bone quality of the elderly patient.^2^ Studies have shown that age has no effect on clinical score and if the stem has a good press-fit, the chances for an excellent result with bone ingrowth is very good.^11^ Better understanding of the mechanism and interface has led surgeons to prefer cementless femoral fixation even in the osteoporotic bone of the very elderly,^6^,^11^,^13^ with 100% survival rates for both the cup and stem in large series of >80-year-old patients.^21^,^22^ A word of caution has however been expressed by Ogino *et al*., who concluded that cementless total hip arthroplasty in patients aged 80 years or more showed higher incidence of dislocation, periprosthetic fracture, and infection.^23^

In our experience, also, we observed that a tight fit of the implant is easily achieved even in osteoporotic bone. Though cementless stems are not preferred in very wide femoral canals (Dorr types C and D),^2^ we now have available improved stem designs (with metaphyseal flares and larger stem diameters, combined proximal and distal fixation, etc., e.g., the Corail^™^ and Solution^™^ stems) that permit press-fit even in these femora. Perhaps, this modular interface made cementless fixation possible in all our (intra- and extracaspular fractures) patients.

Prior to starting our study, we had experienced two instances of intraoperative cardiac arrest (in patients with cardiac comorbidity) while cementing the femoral stem. Although it can be argued that there may have been many reasons for these two deaths (preexisting cardiac morbidity, ASA grade of risk, anesthetist competence, unavailability of cardiologists in the vicinity of the operating room, etc.), the experience prompted us to consider cementless femoral fixation in the very elderly with associated multiple comorbidities. We have now come to prefer cementless femoral fixation in these patients. Although we progressed gradually from toe-touch to full weight-bearing in our series of patients, stem subsidence or thigh pain has not been seen in the immediate (3 months) or last (average: 36 months) follow-up. Our patients had achieved preinjury ambulatory status at 3 months.

The relatively low morbidity and encouraging short-term clinical results in this series lead us to recommend cementless bipolar arthroplasty as an option of treatment in these very elderly patients with femoral neck fractures and multiple comorbidities. Our study is, however, limited by the small number of subjects and the short follow-up.
